# Flux syntheses and single-crystal structures of CsNa_10_ *M*_4_(AsO_4_)_9_ (*M* = Zr, Hf)

**DOI:** 10.1107/S2056989022006338

**Published:** 2022-06-21

**Authors:** William T. A. Harrison

**Affiliations:** a Department of Chemistry, University of Aberdeen, Meston Walk, Aberdeen AB24 3UE, Scotland

**Keywords:** crystal structure, zirconium, hafnium, arsenate, ionic conduction

## Abstract

The isostructural compounds caesium deca­sodium tetra­zirconium nona­arsenate, CsNa_10_Zr_4_(AsO_4_)_9_, and caesium deca­sodium tetra­hafnium nona­arsenate, CsNa_10_Hf_4_(AsO_4_)_9_, arose as unexpected single-crystal products from the reactions of Na_2_CO_3_, MO_2_ (*M* = Zr, Hf) and As_2_O_5_ in a eutectic flux of NaCl and CsCl. They consist of *M*O_6_ octa­hedra and AsO_4_ tetra­hedra sharing vertices to generate three-dimensional polyhedral networks encapsulating the caesium and sodium ions. The MO_6_ groups share all their vertices with adjacent As atoms but the As atoms have one or two ‘terminal’ O atoms not bonded to Zr or Hf. The Cs^+^ ion adopts a squashed octa­hedral geometry and the coordination polyhedra of the partially occupied sodium ions are variously trigonal bipyramidal, tetra­hedral, square pyramidal and trigonal pyramidal. Site symmetries: Cs  ; *M* 3; As 1 and 2; O 1; Na 1, 2 and 3. The *M* = Zr crystal was refined as an obverse/reverse rhombohedral twin.

## Abstract 2

The isostructural title compounds arose as unexpected single-crystal products from the reactions of Na_2_CO_3_, MO_2_ (*M* = Zr, Hf) and As_2_O_5_ in a NaCl/CsCl eutectic flux.

## 1. Chemical context

Potassium titanyl phosphate (KTiOPO_4_; KTP) has long been recognized as an important non-linear optical (NLO) material (Zumsteg *et al.*, 1976 ▸) due to its unique combination of desirable physical properties including ‘a large hyperpolarizability, excellent temperature window, wide wavelength for phase matching and outstanding crystal stability’ (Stucky *et al.*, 1989 ▸). Work continues to improve the performance of KTP waveguides in optoelectronics (Kores *et al.*, 2021 ▸) and it is finding new uses as a frequency doubler (to 532 nm green light) for 1064 nm Nd–YAG laser radiation in many areas of medicine (Shim & Kim, 2021 ▸; McGarey *et al.*, 2021 ▸). So far as crystal chemistry is concerned, the KTiOPO_4_ structure type (space group *Pna*2_1_, *a* ≃ 12.8, *b* ≃ 6.4, *c* ≃ 10.6 Å, *Z* = 8, *Z*′ = 2) is remarkably accommodating with respect to partial or complete isovalent or aleovalent substitution at the potassium (Na^+^, Rb^+^, Cs^+^, Tl^+^, NH_4_^+^…), titanium (Zr^IV^, Hf^IV^, V^IV^, Sn^IV^, Sb^V^, Ga^3+^, Fe^3+^, Al^3+^, Cr^3+^…), phospho­rus (As^V^, Si^IV^, Ge^IV^) and even oxygen (OH^−^, F^−^) sites and comprehensive reviews on its substitution chemistry have appeared (Sorokina & Voronkova, 2007 ▸).

In an attempt to grow single crystals of the possible new KTP analogues NaZrOAsO_4_ and NaHfOAsO_4_ by reacting Na_2_CO_3_, *M*O_2_ (*M* = Zr, Hf) and As_2_O_5_ in a low-melting flux of NaCl and CsCl, the isostructural title compounds CsNa_10_Zr_4_(AsO_4_)_9_ (I) and CsNa_10_Hf_4_(AsO_4_)_9_ (II) were the unexpected result and their crystal structures are now described.

## 2. Structural commentary

Compounds (I) and (II) are isostructural and crystallize in the rhombohedral space group *R* 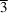 *c* (No. 167) with an unusually long *c* unit-cell parameter of nearly 77 Å. This is of course partly a consequence of our choosing the hexa­gonal (*R*-centred) setting of the unit cell [the equivalent primitive rhombohedral lattice for (I) has *a* = *b* = *c* ≃ 26.21 Å and α = β = γ ≃ 20.3°] but even so, it is notable that the *l* index runs well into three figures for (I) in the *R*-centred setting. This description will focus on the structure of (I) and note significant differences for (II) where applicable.

**Figure 1:**
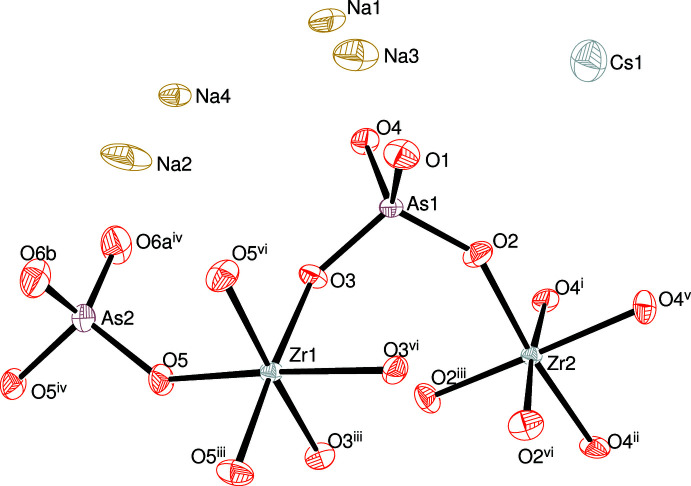
The asymmetric unit of (I) expanded to include the full Zr and As coordination polyhedra showing 50% displacement ellipsoids. Only one disorder component about As2 is shown. Symmetry codes: (i) 1 − *x*, 1 − *y*, −*z*; (ii) *y*, 1 − *x* − *y*, −*z*; (iii) 1 − *y*, 1 + *x* − *y*, *z*; (iv) 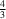 − *x*, 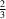 − *x* + *y*, 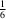 − *z*; (v) *x* − y, *x*, −z; (vi) *y* − x, 1 − *x*, *z*.

The asymmetric unit of (I), expanded to show the full coordination polyhedra of the zirconium and arsenic atoms, is shown in Fig. 1 ▸. It consists of two zirconium atoms (both with site symmetry 3 on Wyckoff site 12*c*), two arsenic atoms [As1 on a general position (36*f*) and As2 with site symmetry 2 (18*e*)] and six oxygen atoms, one of which is disordered over two adjacent sites (all lying on general positions, 36*f*), which leads to the unusual 4:9 stoichiometry for the Zr^IV^ and AsO_4_^3–^ moieties with a net charge of −11. The structure of (I) is completed by a Cs^+^ ion (site symmetry 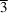 , 6*b*) and four partly occupied sodium cations [one on a general position (36*f*), one with site symmetry 2 (18*e*) and two with site symmetry 3 (12*c*)]. To maintain charge balance, the four sodium ions must have a total occupancy of 10 based on *Z* = 6 (full occupancy of the four sites would give 13 sodium ions per caesium ion).

Both zirconium atoms adopt almost regular ZrO_6_ octa­hedral geometries (Müller-Buschbaum, 2010 ▸) when crystal symmetry is taken into account: the mean Zr1—O separation (to 3 × O3 and 3 × O5) is 2.070 Å and the quadratic elongation and angular variance are 1.001 and 4.43°^2^, respectively (Robinson *et al.*, 1971 ▸). Equivalent data for Zr2 (bonded to to 3 × O2 and 3 × O4) are 2.072 Å, 1.003 and 9.86°^2^, respectively. The ‘extrapolated’ (Brese & O’Keeffe, 1991 ▸) bond-valence sums (BVS) in valence units are 4.10 and 4.07 for Zr1 and Zr2, respectively, in acceptable agreement with the expected value of 4.00. The mean Hf—O distances in (II) are 2.062 Å for Hf1 (BVS = 4.13, quadratic elongation = 1.002, angular variance = 5.38°^2^) and 2.065 Å for Hf2 (4.10, 1.004, 13.20°^2^). It may be seen that the Hf—O bonds are slightly shorter than the Zr—O bonds, which is in accordance with ionic radii data (Shannon, 1976 ▸): *r*_6_(Zr^IV^) = 0.72 (6 = six-coordinate) and *r*_6_(Hf^IV^) = 0.71 Å and is presumed to arise from the lanthanide contraction effect.

The As1 atom in (I) is surrounded by four oxygen atoms (O1–O4) in the geometry of a slightly distorted tetra­hedron [mean As—O = 1.677 Å, spread of O—As—O angles = 103.0 (2)–114.9 (2)°, τ_4_ (Yang *et al.*, 2007 ▸) = 0.95]. Atom As2 is also tetra­hedral (to 2 × O5 and 2 × O6), with the latter O atom disordered over two adjacent sites in almost equal occupancies of 0.45 (3):0.55 (3) [O6*A*⋯O6*B* = 0.909 (13) Å]. Of the six oxygen atoms in the structure of (I), four of them (O2–O5) bridge zirconium and arsenic atoms with a mean Zr—O—As bond angle of 141.5° [equivalent mean Hf—O—As bond angle in (II) = 140.4°] and two (O1 and O6) are ‘terminal’ and only bonded to arsenic: all of the O atoms also form one or more bonds to nearby caesium and/or sodium ions.

The caesium ion in (I) adopts a grossly squashed octa­hedral coordination to six O1 atoms with Cs1—O1 = 3.235 (4) Å: the *cis* O—Cs—O bond angles are compressed to 62.30 (10) or expanded to 117.70 (10)°: the Cs1 BVS of 0.61 compared to an expected value of 1.00 suggests significant underbonding. The inter­pretation of the sodium-ion coordination polyhedra are complicated by the positional disorder of atom O6 but can be described as distorted trigonal bipyramidal (Na1), very distorted tetra­hedral (Na2), square-based pyramidal (Na3) and squashed trigonal pyramidal (Na4). It is notable that Na4 is only three coordinate but similar NaO_3_ geometries have been observed in dehydrated sodium aluminosilicate zeolites (Adams *et al.*, 1982 ▸).

**Figure 2:**
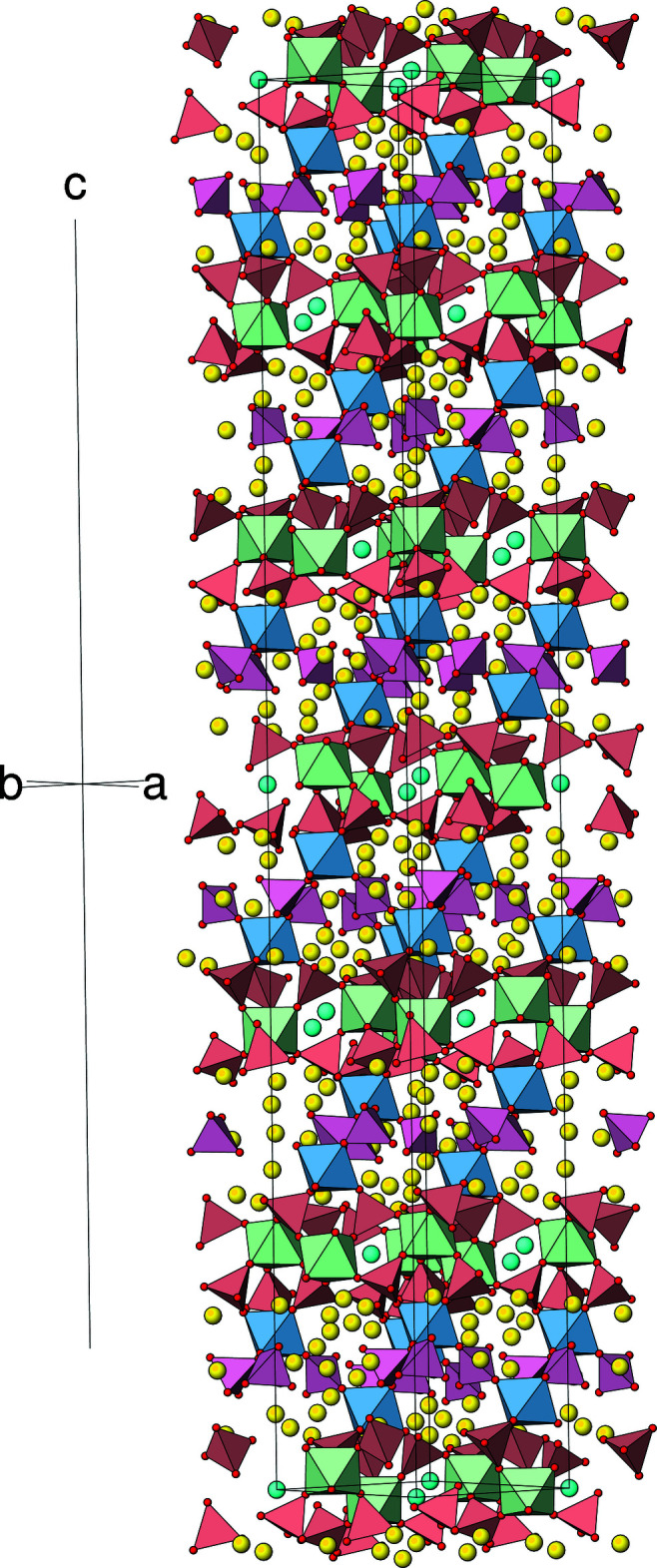
The unit-cell of (I) in polyhedral representation viewed approximately down [110]. A single O atom at the average location of O6*A* and O6*B* in the asymmetric unit has been used to construct the As2 tetra­hedron. Colour code: Zr1O_6_ octa­hedra blue, Zr2O_6_ octa­hedra green, As1O_4_ tetra­hedra peach, As2O_4_ tetra­hedra rose, Cs sky blue, Na yellow, O (polyhedral corners) red.

**Figure 3:**
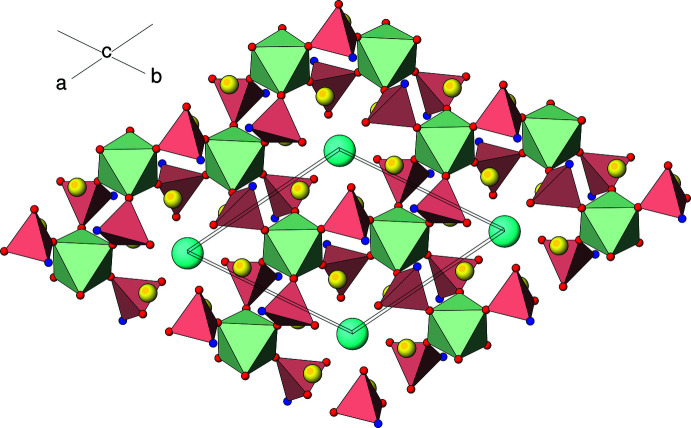
View down [001] of an ‘A’-type layer in the structure of (I) in polyhedral representation. Atom and polyhedron colours as in Fig. 2 ▸ except O3 is blue.

**Figure 4:**
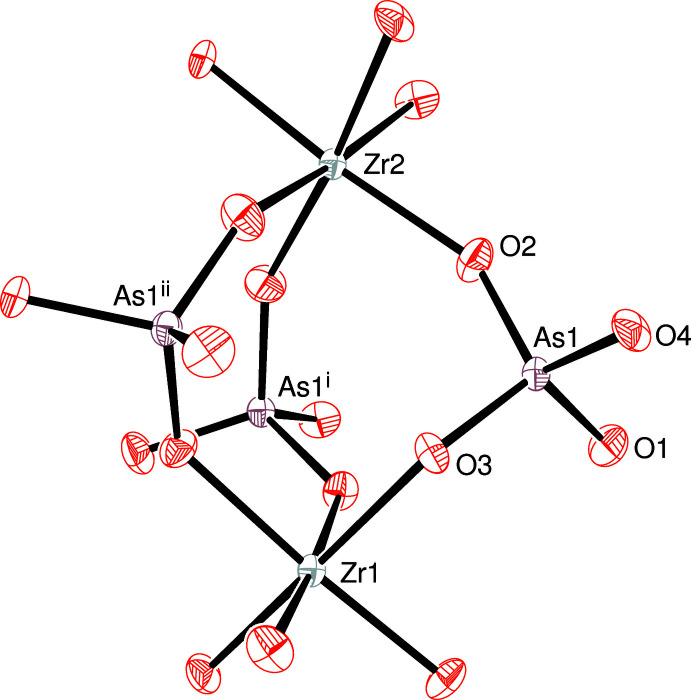
Detail of the extended structure of (I) showing a Zr_2_As_3_O_18_ ‘lantern’ motif of Zr1 and Zr2 octa­hedra linked by three As1 tetra­hedra *via* atoms O2 and O3. In (I), this motif has crystallographically imposed threefold symmetry about a rotation axis passing through the zirconium atoms. Symmetry codes: (i) 1 − *y*, 1 + *x* − *y*, *z*; (ii) *y* − *x*, 1 − *x*, *z*.

The extended structure of (I) (Fig. 2 ▸) can be conceptually broken down into two different types of layers lying parallel to (001). The first layer (type ‘A’) occurs at *z* ≃ 0, 1/6, 1/3, 1/2, 2/3 and 5/6 with adjacent A-layers laterally displaced by 1/3 in *x* and 2/3 in *y* and consists of the Zr2 and As1 centred polyhedra as well as the caesium ions. Fig. 3 ▸ shows that each Zr2O_6_ octa­hedron is connected by two As1O_4_ tetra­hedra (*via* O2 and O4) to result in a ‘honeycomb’ array of polyhedral 12-rings (six octa­hedra and 12 tetra­hedra) encapsulating the Cs^+^ ions. Atom O3 of the arsenate group provides the link to the type ‘B’ layers on either side of the A layer. This inter-octa­hedral connectivity *via* O3 leads to a distinctive ‘lantern’ motif (Fig. 4 ▸) in which three tetra­hedra link two octa­hedra [Zr1⋯Zr2 = 4.886 (2); Hf1⋯Hf2 in (II) = 4.863 (2) Å]: similar ‘lanterns’ are a feature of the polyhedral connectivity in the scandium tungstate [*M*_2_(*X*O_4_)_3_] (Abrahams & Bernstein, 1966 ▸), Nasicon [*AM*_2_(*X*O_4_)_3_] (Anantharamulu *et al.*, 2011 ▸) and langbeinite [*A*_2_ *M*_2_(*X*O_4_)_3_] (Norberg, 2002 ▸) structure types but they differ from (I) because all the vertices of the constituent tetra­hedra in these structures link to adjacent octa­hedra, hence their 2:3 *M*:*X* ratios compared to the 4:9 ratio for (I).

**Figure 5:**
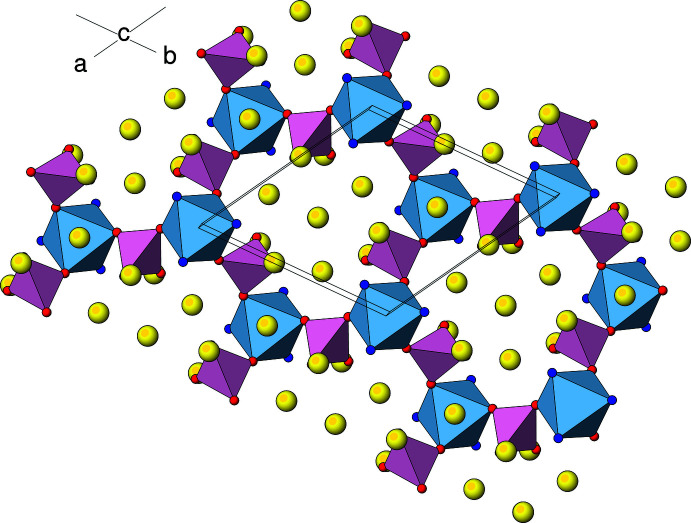
View down [001] of a ‘B’-type layer in the structure of (I) in polyhedral representation. Atom and polyhedron colours as in Fig. 2 ▸ except O3 is blue.

The B layers in (I) (Fig. 5 ▸) lie at *z* ≃ 1/12, 1/4, 5/12, 7/12, 3/4 and 11/12 and are associated with the Zr1 and As2 species. These also feature polyhedral 12-rings (six octa­hedra and six tetra­hedra) but only one As2 tetra­hedron (with two terminal As2—O6 bonds) links adjacent Zr1 octa­hedra *via* atom O5. There are numerous sodium sites associated with the B layers. The disorder of the sodium ions in the vicinities of the B layers and possible small [110] channels (see Fig. 2 ▸) suggests the possibility of ionic conductivity (Norberg, 2002 ▸). An analysis of the stucture with *PLATON* (Spek, 2020 ▸) with the sodium ions removed indicated that there was 119.4 Å^3^ of free space per unit cell (∼2.1%).

## 3. Database survey

A survey of the Inorganic Crystal Structure Database (ICSD) (Belsky *et al.*, 2002 ▸) revealed 11 matches for crystal structures containing Zr + As + O, the majority of these being Nasicon (Anantharamulu *et al.*, 2011 ▸) derivatives such as NaZr_2_(AsO_4_)_3_ (Chakir *et al.*, 2003 ▸) or KZr_2_(AsO_4_)_3_ (Elbrahimi & Durand, 1990 ▸) as well as one KTP analogue, *viz*. RbZrOAsO_4_ (Simpson & Harrison, 2004 ▸). There were no hits for the combination of Hf + As + O.

## 4. Synthesis and crystallization

Compound (I) was prepared by mixing 1.00 g of Na_2_CO_3_, 0.581 g of ZrO_2_ and 1.399 g of As_2_O_5_ (Na:Zr:As molar ratio ≃ 4:1:3) in an agate mortar: 1.00 g of this mixture was added to 3.0 g of a eutectic-melt mixture (*T*_melt_ ≃ 500°C) of NaCl/CsCl (∼0.35:0.65 mol) and placed in a flat-bottom alumina crucible. The crucible was rapidly heated to 500°C in a muffle furnace and then ramped at 12°C min^−1^ to 700°C and cooled at the same rate to 400°C and then removed from the furnace and left to cool. The gummy white product was washed with copious amounts of hot water followed by acetone to result in a mass of tiny colourless rods of (I). Compound (II) was made in the same way starting from a pre-mixture of 1.00 g Na_2_CO_3_, 1.12 g HfO_2_ and 1.57 g As_2_O_5_ and tiny colourless rods of (II) were the result.

Caution! Arsenic compounds are highly toxic and carcinogenic. Take all appropriate safety precautions, especially with respect to dust contamination.

## 5. Refinement

Crystal data, data collection and structure refinement details are summarized in Table 1 ▸. The crystal chosen for data collection for (I) was found to be twinned over its rhombohedral obverse and reverse settings (Herbst-Irmer & Sheldrick, 2002 ▸) in a 0.797 (3):0.203 (3) ratio, which was processed as a *SHELXL* HKLF 5 refinement. To ensure charge balance, the occupancies of the four partially occupied sodium sites must sum to 10.0 Na per caesium ion and this was achieved by using a SUMP card (linear free variable restraint) in *SHELXL*, as unrestrained refinements tended to drift to a collective occupancy of above 10 (full occupancy of the four sodium sites would give 13 Na to 1 Cs). This needed cautious damped refinement cycles to begin with, but as the refinement converged, the damping could be removed to give refined fractional site occupancies of Na1 = 0.852 (5), Na2 = 0.860 (9), Na3 = 0.731 (12) and Na4 = 0.423 (11) for (I) and Na1 = 0.887 (7), Na2 = 0.846 (11), Na3 = 0.735 (16) and Na4 = 0.337 (14) for (II). The final difference map for (II) features electron density peaks of ∼2 e Å^−3^ near some of the sodium ions, perhaps suggesting that they are localizing over split multiple sites at low temperatures, but efforts to model this did not lead to satisfactory refinements. The value of *U*_eq_ for Na4 is small, which might indicate partial occupancy of caesium on this site (*i.e.*, a formula of Cs_1+x_Na_10-x_Hf_4_(AsO_4_)_9_, but attempts to model this were inconclusive.

## Supplementary Material

Crystal structure: contains datablock(s) I, II, global. DOI: 10.1107/S2056989022006338/pk2665sup1.cif

Structure factors: contains datablock(s) I. DOI: 10.1107/S2056989022006338/pk2665Isup2.hkl

Structure factors: contains datablock(s) II. DOI: 10.1107/S2056989022006338/pk2665IIsup3.hkl

CCDC references: 2179420, 2179419

Additional supporting information:  crystallographic information; 3D view; checkCIF report

## Figures and Tables

**Table 1:** Experimental details

	(I)	(II)
Crystal data		
Chemical formula	CsNa_10_Zr_4_(AsO_4_)_9_	CsNa_10_Hf_4_(AsO_4_)_9_
*M*_r_	1977.97	2327.05
Crystal system, space group	Trigonal, *R* 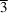 *c*:*H*	Trigonal, *R* 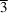 *c*:*H*
Temperature (K)	293	120
*a*, *c* (Å)	9.2218 (5), 76.982 (5)	9.1795 (2), 76.527 (8)
*V* (Å^3^)	5669.6 (7)	5584.5 (6)
*Z*	6	6
Radiation type	Mo *K*α	Mo *K*α
μ (mm^−1^)	10.07	20.25
Crystal size (mm)	0.10 × 0.10 × 0.10	0.08 × 0.08 × 0.08

Data collection		
Diffractometer	Bruker SMART CCD	Nonius KappaCCD
Absorption correction	Multi-scan (*SADABS*; Bruker, 1999 ▸)	Multi-scan (*SORTAV*; Blessing, 1995 ▸)
*T*_min_, *T*_max_	0.350, 0.495	0.40, 0.50
No. of measured, independent and observed [*I* > 2σ(*I*)] reflections	2288, 2288, 1694	11660, 1434, 1164
*R*_int_	–	0.070
(sin θ/λ)_max_ (Å^−1^)	0.756	0.650

Refinement		
*R*[*F* ^2^ > 2σ(*F* ^2^)], *wR*(*F* ^2^), *S*	0.040, 0.093, 1.00	0.032, 0.078, 1.06
No. of reflections	2288	1434
No. of parameters	112	107
No. of restraints	1	7
Δρ_max_, Δρ_min_ (e Å^−3^)	1.85, −1.63	2.57, −2.00

Computer programs: *SMART* and *SAINT* (Bruker, 1999 ▸), *DENZO*/*SCALEPACK* (Otwinowski & Minor, 1997 ▸), *SHELXS* and *SHELXS97* (Sheldrick, 2008 ▸), *SHELXL2018/3* (Sheldrick, 2015 ▸), *ORTEP-3 for Windows* (Farrugia, 2012 ▸), *ATOMS* (Dowty, 2005 ▸) and *publCIF* (Westrip, 2010 ▸).

## supplementary crystallographic information

### Caesium decasodium tetrazirconium nonaarsenate (I) Crystal data

**Table:**

CsNa_10_Zr_4_(AsO_4_)_9_	*D*_x_ = 3.476 Mg m^−^^3^
*M**_r_* = 1977.97	Mo *K*α radiation, λ = 0.71073 Å
Trigonal, *R*3*c*:*H*	Cell parameters from 2999 reflections
*a* = 9.2218 (5) Å	θ = 2.6–30.8°
*c* = 76.982 (5) Å	µ = 10.07 mm^−^^1^
*V* = 5669.6 (7) Å^3^	*T* = 293 K
*Z* = 6	Prism, colourless
*F*(000) = 5460	0.10 × 0.10 × 0.10 mm

### Caesium decasodium tetrazirconium nonaarsenate (I) Data collection

**Table:**

Bruker SMART CCD diffractometer	1694 reflections with *I* > 2σ(*I*)
ω scans	θ_max_ = 32.5°, θ_min_ = 2.6°
Absorption correction: multi-scan (SADABS; Bruker, 1999)	*h* = −12→12
*T*_min_ = 0.350, *T*_max_ = 0.495	*k* = −13→13
2288 measured reflections	*l* = 0→114
2288 independent reflections	

### Caesium decasodium tetrazirconium nonaarsenate (I) Refinement

**Table:**

Refinement on *F*^2^	1 restraint
Least-squares matrix: full	Primary atom site location: structure-invariant direct methods
*R*[*F*^2^ > 2σ(*F*^2^)] = 0.040	*w* = 1/[σ^2^(*F*_o_^2^) + (0.0452*P*)^2^] where *P* = (*F*_o_^2^ + 2*F*_c_^2^)/3
*wR*(*F*^2^) = 0.093	(Δ/σ)_max_ < 0.001
*S* = 1.00	Δρ_max_ = 1.85 e Å^−^^3^
2288 reflections	Δρ_min_ = −1.63 e Å^−^^3^
112 parameters	

### Caesium decasodium tetrazirconium nonaarsenate (I) Special details

**Table:**

Geometry. All esds (except the esd in the dihedral angle between two l.s. planes) are estimated using the full covariance matrix. The cell esds are taken into account individually in the estimation of esds in distances, angles and torsion angles; correlations between esds in cell parameters are only used when they are defined by crystal symmetry. An approximate (isotropic) treatment of cell esds is used for estimating esds involving l.s. planes.
Refinement. Refined as a 2-component obverse/reverse twin.

### Caesium decasodium tetrazirconium nonaarsenate (I) Fractional atomic coordinates and isotropic or equivalent isotropic displacement parameters (Å^2^)

**Table:**

	*x*	*y*	*z*	*U*_iso_*/*U*_eq_	Occ. (<1)
Cs1	0.000000	0.000000	0.000000	0.0381 (2)	
Na1	0.3350 (5)	0.0681 (4)	0.04049 (4)	0.0410 (9)	0.852 (5)
Na2	0.4054 (5)	0.333333	0.083333	0.0411 (13)	0.860 (9)
Na3	0.000000	0.000000	0.05355 (8)	0.0321 (17)	0.731 (12)
Na4	0.666667	0.333333	0.06014 (12)	0.021 (2)	0.423 (11)
Zr1	0.333333	0.666667	0.05681 (2)	0.01237 (15)	
Zr2	0.333333	0.666667	−0.00666 (2)	0.01204 (15)	
As1	0.34556 (6)	0.39774 (6)	0.02640 (2)	0.01350 (11)	
As2	0.666667	0.73010 (9)	0.083333	0.0309 (2)	
O1	0.1757 (5)	0.2319 (4)	0.03370 (5)	0.0251 (8)	
O2	0.3084 (5)	0.4671 (5)	0.00779 (5)	0.0251 (8)	
O3	0.4373 (5)	0.5591 (4)	0.04062 (5)	0.0192 (7)	
O4	0.4946 (5)	0.3445 (5)	0.02328 (5)	0.0217 (8)	
O5	0.5457 (5)	0.7825 (5)	0.07152 (5)	0.0234 (8)	
O6A	0.603 (2)	0.5691 (13)	0.09411 (18)	0.036 (5)	0.45 (3)
O6B	0.5238 (19)	0.5873 (11)	0.09900 (16)	0.034 (4)	0.55 (3)

### Caesium decasodium tetrazirconium nonaarsenate (I) Atomic displacement parameters (Å^2^)

**Table:**

	*U*^11^	*U*^22^	*U*^33^	*U*^12^	*U*^13^	*U*^23^
Cs1	0.0436 (4)	0.0436 (4)	0.0271 (4)	0.02182 (18)	0.000	0.000
Na1	0.072 (2)	0.0286 (15)	0.0375 (16)	0.0369 (17)	−0.0102 (17)	−0.0033 (13)
Na2	0.0307 (18)	0.0138 (18)	0.073 (3)	0.0069 (9)	−0.0044 (10)	−0.009 (2)
Na3	0.0216 (19)	0.0216 (19)	0.053 (4)	0.0108 (9)	0.000	0.000
Na4	0.015 (3)	0.015 (3)	0.034 (5)	0.0074 (14)	0.000	0.000
Zr1	0.0109 (2)	0.0109 (2)	0.0153 (3)	0.00546 (10)	0.000	0.000
Zr2	0.0109 (2)	0.0109 (2)	0.0144 (3)	0.00543 (10)	0.000	0.000
As1	0.0135 (2)	0.0114 (2)	0.0162 (2)	0.00670 (18)	0.00020 (18)	0.00004 (18)
As2	0.0460 (5)	0.0276 (3)	0.0254 (4)	0.0230 (3)	−0.0191 (4)	−0.0095 (2)
O1	0.0227 (19)	0.0166 (17)	0.029 (2)	0.0041 (15)	0.0059 (16)	0.0051 (16)
O2	0.029 (2)	0.0199 (18)	0.0268 (19)	0.0131 (17)	−0.0026 (17)	0.0055 (16)
O3	0.0199 (17)	0.0165 (16)	0.0220 (17)	0.0097 (14)	0.0002 (15)	−0.0050 (15)
O4	0.0246 (19)	0.0293 (19)	0.0227 (18)	0.0219 (17)	0.0003 (15)	−0.0034 (17)
O5	0.0178 (17)	0.028 (2)	0.0252 (18)	0.0123 (16)	−0.0095 (15)	−0.0076 (17)
O6A	0.034 (8)	0.024 (5)	0.034 (6)	0.001 (4)	−0.010 (6)	0.003 (4)
O6B	0.032 (7)	0.021 (4)	0.032 (5)	0.000 (4)	−0.014 (5)	0.004 (4)

### Caesium decasodium tetrazirconium nonaarsenate (I) Geometric parameters (Å, º)

**Table:**

Cs1—O1^i^	3.235 (4)	Na4—O6A^vi^	2.694 (17)
Cs1—O1^ii^	3.235 (4)	Na4—O6A^xii^	2.694 (17)
Cs1—O1^iii^	3.235 (4)	Zr1—O5	2.041 (3)
Cs1—O1	3.235 (4)	Zr1—O5^xiii^	2.041 (3)
Cs1—O1^iv^	3.235 (4)	Zr1—O5^ix^	2.041 (3)
Cs1—O1^v^	3.235 (4)	Zr1—O3^ix^	2.099 (3)
Na1—O6B^vi^	2.193 (9)	Zr1—O3^xiii^	2.099 (3)
Na1—O3^vii^	2.394 (5)	Zr1—O3	2.099 (3)
Na1—O1^v^	2.482 (5)	Zr2—O2^xiii^	2.062 (4)
Na1—O6A^vi^	2.485 (16)	Zr2—O2^ix^	2.062 (4)
Na1—O4	2.582 (5)	Zr2—O2	2.062 (4)
Na1—O1	2.632 (5)	Zr2—O4^xiv^	2.081 (3)
Na2—O6A	2.185 (10)	Zr2—O4^iv^	2.081 (3)
Na2—O6A^vi^	2.185 (10)	Zr2—O4^xv^	2.081 (3)
Na2—O6B	2.361 (12)	As1—O1	1.647 (4)
Na2—O6B^vi^	2.361 (12)	As1—O2	1.673 (4)
Na2—O5^viii^	2.495 (5)	As1—O4	1.691 (3)
Na2—O5^ix^	2.495 (5)	As1—O3	1.694 (4)
Na3—O1^ii^	2.463 (5)	As2—O6A	1.538 (10)
Na3—O1	2.463 (5)	As2—O6A^xii^	1.538 (10)
Na3—O1^v^	2.463 (5)	As2—O5^xii^	1.686 (4)
Na3—O6B^viii^	2.47 (2)	As2—O5	1.686 (4)
Na3—O6B^x^	2.47 (2)	As2—O6B	1.786 (13)
Na3—O6B^vi^	2.47 (2)	As2—O6B^xii^	1.786 (13)
Na4—O6A^xi^	2.694 (17)	O6A—O6B	0.909 (13)
			
O1^i^—Cs1—O1^ii^	180.0 (3)	O5^xiii^—Zr1—O3^ix^	87.67 (16)
O1^i^—Cs1—O1^iii^	62.30 (10)	O5^ix^—Zr1—O3^ix^	91.79 (15)
O1^ii^—Cs1—O1^iii^	117.70 (10)	O5—Zr1—O3^xiii^	87.66 (16)
O1^i^—Cs1—O1	117.70 (10)	O5^xiii^—Zr1—O3^xiii^	91.79 (15)
O1^ii^—Cs1—O1	62.30 (10)	O5^ix^—Zr1—O3^xiii^	176.01 (15)
O1^iii^—Cs1—O1	180.00 (9)	O3^ix^—Zr1—O3^xiii^	88.35 (14)
O1^i^—Cs1—O1^iv^	62.30 (10)	O5—Zr1—O3	91.79 (15)
O1^ii^—Cs1—O1^iv^	117.70 (10)	O5^xiii^—Zr1—O3	176.01 (15)
O1^iii^—Cs1—O1^iv^	62.30 (10)	O5^ix^—Zr1—O3	87.67 (16)
O1—Cs1—O1^iv^	117.70 (10)	O3^ix^—Zr1—O3	88.35 (14)
O1^i^—Cs1—O1^v^	117.70 (10)	O3^xiii^—Zr1—O3	88.35 (14)
O1^ii^—Cs1—O1^v^	62.30 (10)	O2^xiii^—Zr2—O2^ix^	93.66 (15)
O1^iii^—Cs1—O1^v^	117.70 (10)	O2^xiii^—Zr2—O2	93.66 (15)
O1—Cs1—O1^v^	62.30 (10)	O2^ix^—Zr2—O2	93.66 (15)
O1^iv^—Cs1—O1^v^	180.00 (16)	O2^xiii^—Zr2—O4^xiv^	92.00 (16)
O6B^vi^—Na1—O3^vii^	104.6 (4)	O2^ix^—Zr2—O4^xiv^	88.01 (15)
O6B^vi^—Na1—O1^v^	93.4 (5)	O2—Zr2—O4^xiv^	173.98 (15)
O3^vii^—Na1—O1^v^	87.41 (15)	O2^xiii^—Zr2—O4^iv^	173.98 (15)
O6B^vi^—Na1—O6A^vi^	21.3 (3)	O2^ix^—Zr2—O4^iv^	92.00 (16)
O3^vii^—Na1—O6A^vi^	90.3 (4)	O2—Zr2—O4^iv^	88.01 (15)
O1^v^—Na1—O6A^vi^	108.7 (4)	O4^xiv^—Zr2—O4^iv^	86.15 (14)
O6B^vi^—Na1—O4	118.5 (3)	O2^xiii^—Zr2—O4^xv^	88.01 (15)
O3^vii^—Na1—O4	119.01 (18)	O2^ix^—Zr2—O4^xv^	173.98 (15)
O1^v^—Na1—O4	127.39 (17)	O2—Zr2—O4^xv^	92.01 (16)
O6A^vi^—Na1—O4	115.1 (3)	O4^xiv^—Zr2—O4^xv^	86.15 (14)
O6B^vi^—Na1—O1	85.3 (5)	O4^iv^—Zr2—O4^xv^	86.15 (14)
O3^vii^—Na1—O1	165.74 (19)	O1—As1—O2	111.5 (2)
O1^v^—Na1—O1	81.68 (19)	O1—As1—O4	108.17 (19)
O6A^vi^—Na1—O1	101.8 (5)	O2—As1—O4	109.94 (19)
O4—Na1—O1	62.47 (13)	O1—As1—O3	114.88 (19)
O6B^vi^—Na1—O4^vii^	132.4 (6)	O2—As1—O3	108.98 (18)
O3^vii^—Na1—O4^vii^	58.02 (13)	O4—As1—O3	103.03 (18)
O1^v^—Na1—O4^vii^	125.80 (15)	O6A—As2—O6A^xii^	78.4 (18)
O6A^vi^—Na1—O4^vii^	111.4 (6)	O6A—As2—O5^xii^	112.1 (4)
O4—Na1—O4^vii^	61.11 (16)	O6A^xii^—As2—O5^xii^	125.4 (7)
O1—Na1—O4^vii^	122.35 (15)	O6A—As2—O5	125.4 (7)
O6A—Na2—O6A^vi^	140.7 (13)	O6A^xii^—As2—O5	112.1 (4)
O6A—Na2—O6B	22.7 (4)	O5^xii^—As2—O5	103.8 (3)
O6A^vi^—Na2—O6B	161.0 (11)	O6A—As2—O6B	30.6 (6)
O6A—Na2—O6B^vi^	161.0 (11)	O6A^xii^—As2—O6B	106.8 (15)
O6A^vi^—Na2—O6B^vi^	22.7 (4)	O5^xii^—As2—O6B	103.3 (4)
O6B—Na2—O6B^vi^	176.2 (9)	O5—As2—O6B	103.1 (5)
O6A—Na2—O5^viii^	118.7 (7)	O6A—As2—O6B^xii^	106.8 (15)
O6A^vi^—Na2—O5^viii^	95.2 (5)	O6A^xii^—As2—O6B^xii^	30.6 (6)
O6B—Na2—O5^viii^	96.8 (5)	O5^xii^—As2—O6B^xii^	103.0 (5)
O6B^vi^—Na2—O5^viii^	79.9 (4)	O5—As2—O6B^xii^	103.2 (4)
O6A—Na2—O5^ix^	95.2 (5)	O6B—As2—O6B^xii^	136.7 (12)
O6A^vi^—Na2—O5^ix^	118.7 (7)	As1—O1—Na3	157.7 (2)
O6B—Na2—O5^ix^	79.9 (4)	As1—O1—Na1^ii^	117.0 (2)
O6B^vi^—Na2—O5^ix^	96.8 (5)	Na3—O1—Na1^ii^	74.73 (13)
O5^viii^—Na2—O5^ix^	64.2 (2)	As1—O1—Na1	93.24 (18)
O6A—Na2—O6A^vii^	113.6 (6)	Na3—O1—Na1	72.10 (12)
O6A^vi^—Na2—O6A^vii^	40.3 (6)	Na1^ii^—O1—Na1	146.6 (2)
O6B—Na2—O6A^vii^	124.1 (3)	As1—O1—Cs1	105.65 (17)
O6B^vi^—Na2—O6A^vii^	58.3 (4)	Na3—O1—Cs1	91.66 (16)
O5^viii^—Na2—O6A^vii^	92.7 (2)	Na1^ii^—O1—Cs1	93.89 (14)
O5^ix^—Na2—O6A^vii^	149.8 (3)	Na1—O1—Cs1	91.09 (13)
O6A—Na2—O6A^xii^	40.3 (6)	As1—O2—Zr2	148.2 (2)
O6A^vi^—Na2—O6A^xii^	113.6 (6)	As1—O3—Zr1	130.8 (2)
O6B—Na2—O6A^xii^	58.3 (5)	As1—O3—Na1^xvi^	108.79 (18)
O6B^vi^—Na2—O6A^xii^	124.1 (3)	Zr1—O3—Na1^xvi^	120.42 (18)
O5^viii^—Na2—O6A^xii^	149.8 (2)	As1—O4—Zr2^xv^	148.4 (2)
O5^ix^—Na2—O6A^xii^	92.7 (2)	As1—O4—Na1	93.97 (17)
O6A^vii^—Na2—O6A^xii^	114.9 (5)	Zr2^xv^—O4—Na1	109.81 (17)
O1^ii^—Na3—O1	85.6 (2)	As1—O4—Na1^xvi^	87.30 (16)
O1^ii^—Na3—O1^v^	85.6 (2)	Zr2^xv^—O4—Na1^xvi^	96.88 (15)
O1—Na3—O1^v^	85.6 (2)	Na1—O4—Na1^xvi^	121.76 (18)
O1^ii^—Na3—O6B^viii^	83.5 (2)	As2—O5—Zr1	138.4 (2)
O1—Na3—O6B^viii^	87.5 (2)	As2—O5—Na2^xiii^	95.98 (16)
O1^v^—Na3—O6B^viii^	167.5 (3)	Zr1—O5—Na2^xiii^	124.11 (18)
O1^ii^—Na3—O6B^x^	87.5 (2)	As2—O6A—Na2	118.8 (6)
O1—Na3—O6B^x^	167.5 (3)	As2—O6A—Na1^vi^	116.7 (8)
O1^v^—Na3—O6B^x^	83.5 (2)	Na2—O6A—Na1^vi^	115.9 (6)
O6B^viii^—Na3—O6B^x^	102.1 (3)	As2—O6A—Na4^xi^	147.0 (14)
O1^ii^—Na3—O6B^vi^	167.5 (3)	Na2—O6A—Na4^xi^	75.0 (4)
O1—Na3—O6B^vi^	83.5 (2)	Na1^vi^—O6A—Na4^xi^	75.8 (3)
O1^v^—Na3—O6B^vi^	87.5 (2)	As2—O6A—Na2^xvi^	83.8 (9)
O6B^viii^—Na3—O6B^vi^	102.1 (3)	Na2—O6A—Na2^xvi^	106.0 (9)
O6B^x^—Na3—O6B^vi^	102.1 (3)	Na1^vi^—O6A—Na2^xvi^	109.3 (5)
O6A^xi^—Na4—O6A^vi^	108.1 (5)	Na4^xi^—O6A—Na2^xvi^	63.2 (5)
O6A^xi^—Na4—O6A^xii^	108.1 (5)	As2—O6B—Na1^vi^	120.5 (5)
O6A^vi^—Na4—O6A^xii^	108.1 (5)	As2—O6B—Na2	101.0 (7)
O5—Zr1—O5^xiii^	92.21 (16)	Na1^vi^—O6B—Na2	120.8 (4)
O5—Zr1—O5^ix^	92.21 (16)	As2—O6B—Na3^xvii^	116.8 (7)
O5^xiii^—Zr1—O5^ix^	92.20 (16)	Na1^vi^—O6B—Na3^xvii^	79.9 (6)
O5—Zr1—O3^ix^	176.01 (15)	Na2—O6B—Na3^xvii^	118.3 (6)

Symmetry codes: (i) *y*, −*x*+*y*, −*z*; (ii) −*y*, *x*−*y*, *z*; (iii) −*x*, −*y*, −*z*; (iv) *x*−*y*, *x*, −*z*; (v) −*x*+*y*, −*x*, *z*; (vi) *x*−*y*+1/3, −*y*+2/3, −*z*+1/6; (vii) −*y*+1, *x*−*y*, *z*; (viii) *y*−2/3, *x*−1/3, −*z*+1/6; (ix) −*x*+*y*, −*x*+1, *z*; (x) −*x*+1/3, −*x*+*y*−1/3, −*z*+1/6; (xi) *y*+1/3, *x*−1/3, −*z*+1/6; (xii) −*x*+4/3, −*x*+*y*+2/3, −*z*+1/6; (xiii) −*y*+1, *x*−*y*+1, *z*; (xiv) *y*, −*x*+*y*+1, −*z*; (xv) −*x*+1, −*y*+1, −*z*; (xvi) −*x*+*y*+1, −*x*+1, *z*; (xvii) *y*+1/3, *x*+2/3, −*z*+1/6.

### Caesium decasodium tetrahafnium nonaarsenate (II) Crystal data

**Table:**

CsNa_10_Hf_4_(AsO_4_)_9_	*D*_x_ = 4.152 Mg m^−^^3^
*M**_r_* = 2327.05	Mo *K*α radiation, λ = 0.71073 Å
Trigonal, *R*3*c*:*H*	Cell parameters from 22894 reflections
*a* = 9.1795 (2) Å	θ = 2.9–27.5°
*c* = 76.527 (8) Å	µ = 20.25 mm^−^^1^
*V* = 5584.5 (6) Å^3^	*T* = 120 K
*Z* = 6	Prism, colourless
*F*(000) = 6228	0.08 × 0.08 × 0.08 mm

### Caesium decasodium tetrahafnium nonaarsenate (II) Data collection

**Table:**

Nonius KappaCCD diffractometer	1164 reflections with *I* > 2σ(*I*)
ω scans	*R*_int_ = 0.070
Absorption correction: multi-scan (SORTAV; Blessing, 1995)	θ_max_ = 27.5°, θ_min_ = 3.2°
*T*_min_ = 0.40, *T*_max_ = 0.50	*h* = −11→11
11660 measured reflections	*k* = −11→11
1434 independent reflections	*l* = −99→99

### Caesium decasodium tetrahafnium nonaarsenate (II) Refinement

**Table:**

Refinement on *F*^2^	7 restraints
Least-squares matrix: full	Primary atom site location: structure-invariant direct methods
*R*[*F*^2^ > 2σ(*F*^2^)] = 0.032	*w* = 1/[σ^2^(*F*_o_^2^) + (0.0359*P*)^2^ + 136.7342*P*] where *P* = (*F*_o_^2^ + 2*F*_c_^2^)/3
*wR*(*F*^2^) = 0.078	(Δ/σ)_max_ = 0.008
*S* = 1.06	Δρ_max_ = 2.57 e Å^−^^3^
1434 reflections	Δρ_min_ = −2.00 e Å^−^^3^
107 parameters	

### Caesium decasodium tetrahafnium nonaarsenate (II) Special details

**Table:**

Geometry. All esds (except the esd in the dihedral angle between two l.s. planes) are estimated using the full covariance matrix. The cell esds are taken into account individually in the estimation of esds in distances, angles and torsion angles; correlations between esds in cell parameters are only used when they are defined by crystal symmetry. An approximate (isotropic) treatment of cell esds is used for estimating esds involving l.s. planes.

### Caesium decasodium tetrahafnium nonaarsenate (II) Fractional atomic coordinates and isotropic or equivalent isotropic displacement parameters (Å^2^)

**Table:**

	*x*	*y*	*z*	*U*_iso_*/*U*_eq_	Occ. (<1)
Cs1	0.000000	0.000000	0.000000	0.0162 (3)	
Na1	0.3354 (5)	0.0688 (5)	0.04043 (5)	0.0256 (11)	0.887 (7)
Na2	0.4061 (6)	0.333333	0.083333	0.0208 (15)*	0.846 (11)
Na3	0.000000	0.000000	0.05288 (11)	0.018 (2)	0.735 (16)
Na4	0.666667	0.333333	0.06123 (16)	0.002 (4)*	0.337 (14)
Hf1	0.333333	0.666667	0.05673 (2)	0.00710 (15)	
Hf2	0.333333	0.666667	−0.00682 (2)	0.00611 (15)	
As1	0.34520 (9)	0.39770 (9)	0.02639 (2)	0.00578 (18)	
As2	0.666667	0.73244 (13)	0.083333	0.0205 (3)	
O1	0.1730 (6)	0.2332 (6)	0.03369 (6)	0.0113 (11)	
O2	0.3086 (6)	0.4663 (6)	0.00745 (6)	0.0109 (11)	
O3	0.4389 (6)	0.5622 (6)	0.04043 (6)	0.0068 (10)	
O4	0.4922 (6)	0.3405 (6)	0.02356 (6)	0.0097 (11)	
O5	0.5461 (6)	0.7864 (6)	0.07152 (6)	0.0127 (11)	
O6A	0.602 (2)	0.569 (2)	0.0942 (2)	0.008 (2)	0.35 (2)
O6B	0.5276 (16)	0.5861 (12)	0.09848 (14)	0.029 (4)	0.65 (2)

### Caesium decasodium tetrahafnium nonaarsenate (II) Atomic displacement parameters (Å^2^)

**Table:**

	*U*^11^	*U*^22^	*U*^33^	*U*^12^	*U*^13^	*U*^23^
Cs1	0.0181 (4)	0.0181 (4)	0.0125 (5)	0.0091 (2)	0.000	0.000
Na1	0.049 (3)	0.014 (2)	0.023 (2)	0.023 (2)	−0.0094 (18)	−0.0012 (16)
Na3	0.002 (3)	0.002 (3)	0.049 (5)	0.0012 (13)	0.000	0.000
Hf1	0.00580 (19)	0.00580 (19)	0.0097 (3)	0.00290 (9)	0.000	0.000
Hf2	0.00499 (19)	0.00499 (19)	0.0084 (3)	0.00250 (9)	0.000	0.000
As1	0.0056 (4)	0.0037 (4)	0.0086 (3)	0.0027 (3)	0.0002 (3)	0.0002 (3)
As2	0.0312 (7)	0.0178 (4)	0.0170 (6)	0.0156 (4)	−0.0165 (5)	−0.0083 (3)
O1	0.007 (3)	0.007 (3)	0.015 (3)	0.000 (2)	0.001 (2)	0.001 (2)
O2	0.015 (3)	0.009 (3)	0.011 (2)	0.007 (2)	−0.003 (2)	0.000 (2)
O3	0.005 (2)	0.005 (2)	0.011 (2)	0.003 (2)	−0.0006 (19)	−0.0054 (19)
O4	0.010 (3)	0.013 (3)	0.010 (2)	0.010 (2)	0.002 (2)	0.000 (2)
O5	0.007 (3)	0.017 (3)	0.012 (2)	0.004 (2)	−0.006 (2)	−0.006 (2)
O6A	0.008 (2)	0.008 (2)	0.008 (2)	0.0042 (11)	0.00000 (10)	0.00000 (10)
O6B	0.030 (7)	0.021 (5)	0.022 (5)	0.003 (5)	−0.014 (5)	0.002 (4)

### Caesium decasodium tetrahafnium nonaarsenate (II) Geometric parameters (Å, º)

**Table:**

Cs1—O1	3.218 (5)	Na4—O6A^vi^	2.654 (18)
Cs1—O1^i^	3.218 (5)	Na4—O6A^xii^	2.654 (18)
Cs1—O1^ii^	3.218 (5)	Hf1—O5^xiii^	2.039 (5)
Cs1—O1^iii^	3.218 (5)	Hf1—O5^ix^	2.039 (5)
Cs1—O1^iv^	3.218 (5)	Hf1—O5	2.039 (5)
Cs1—O1^v^	3.218 (5)	Hf1—O3^ix^	2.084 (4)
Na1—O6B^vi^	2.212 (10)	Hf1—O3^xiii^	2.084 (4)
Na1—O3^vii^	2.377 (6)	Hf1—O3	2.084 (4)
Na1—O1^iii^	2.443 (6)	Hf2—O2	2.051 (5)
Na1—O6A^vi^	2.467 (17)	Hf2—O2^ix^	2.052 (5)
Na1—O4	2.524 (6)	Hf2—O2^xiii^	2.052 (5)
Na1—O1	2.649 (6)	Hf2—O4^xiv^	2.077 (5)
Na1—O4^vii^	2.972 (6)	Hf2—O4^ii^	2.077 (5)
Na2—O6A	2.170 (16)	Hf2—O4^xv^	2.077 (5)
Na2—O6A^vi^	2.170 (16)	As1—O1	1.644 (5)
Na2—O6B	2.320 (10)	As1—O2	1.680 (5)
Na2—O6B^vi^	2.320 (10)	As1—O4	1.689 (5)
Na2—O5^viii^	2.458 (7)	As1—O3	1.696 (4)
Na2—O5^ix^	2.458 (7)	As2—O6A	1.552 (15)
Na3—O1^v^	2.421 (7)	As2—O6A^xi^	1.552 (15)
Na3—O1^iii^	2.421 (7)	As2—O5	1.684 (5)
Na3—O1	2.421 (7)	As2—O5^xi^	1.684 (5)
Na3—O6B^viii^	2.534 (17)	As2—O6B	1.750 (11)
Na3—O6B^x^	2.534 (17)	As2—O6B^xi^	1.750 (11)
Na3—O6B^vi^	2.534 (17)	O6A—O6B	0.842 (15)
Na4—O6A^xi^	2.654 (18)		
			
O1—Cs1—O1^i^	180.00 (19)	O5^ix^—Hf1—O3	87.7 (2)
O1—Cs1—O1^ii^	117.59 (14)	O5—Hf1—O3	92.3 (2)
O1^i^—Cs1—O1^ii^	62.41 (14)	O3^ix^—Hf1—O3	87.88 (18)
O1—Cs1—O1^iii^	62.41 (14)	O3^xiii^—Hf1—O3	87.88 (18)
O1^i^—Cs1—O1^iii^	117.59 (14)	O2—Hf2—O2^ix^	94.29 (18)
O1^ii^—Cs1—O1^iii^	180.00 (19)	O2—Hf2—O2^xiii^	94.29 (18)
O1—Cs1—O1^iv^	117.59 (14)	O2^ix^—Hf2—O2^xiii^	94.28 (18)
O1^i^—Cs1—O1^iv^	62.41 (14)	O2—Hf2—O4^xiv^	92.38 (19)
O1^ii^—Cs1—O1^iv^	62.41 (14)	O2^ix^—Hf2—O4^xiv^	173.05 (19)
O1^iii^—Cs1—O1^iv^	117.59 (14)	O2^xiii^—Hf2—O4^xiv^	87.19 (19)
O1—Cs1—O1^v^	62.41 (14)	O2—Hf2—O4^ii^	87.18 (19)
O1^i^—Cs1—O1^v^	117.59 (14)	O2^ix^—Hf2—O4^ii^	92.38 (19)
O1^ii^—Cs1—O1^v^	117.59 (14)	O2^xiii^—Hf2—O4^ii^	173.05 (19)
O1^iii^—Cs1—O1^v^	62.41 (14)	O4^xiv^—Hf2—O4^ii^	85.96 (19)
O1^iv^—Cs1—O1^v^	180.0 (3)	O2—Hf2—O4^xv^	173.04 (19)
O6B^vi^—Na1—O3^vii^	103.8 (4)	O2^ix^—Hf2—O4^xv^	87.18 (19)
O6B^vi^—Na1—O1^iii^	94.4 (4)	O2^xiii^—Hf2—O4^xv^	92.38 (19)
O3^vii^—Na1—O1^iii^	86.5 (2)	O4^xiv^—Hf2—O4^xv^	85.96 (19)
O6B^vi^—Na1—O6A^vi^	19.8 (4)	O4^ii^—Hf2—O4^xv^	85.96 (19)
O3^vii^—Na1—O6A^vi^	90.7 (5)	O1—As1—O2	110.9 (3)
O1^iii^—Na1—O6A^vi^	108.7 (5)	O1—As1—O4	108.0 (2)
O6B^vi^—Na1—O4	118.4 (3)	O2—As1—O4	110.3 (2)
O3^vii^—Na1—O4	119.4 (2)	O1—As1—O3	115.4 (2)
O1^iii^—Na1—O4	127.6 (2)	O2—As1—O3	108.7 (2)
O6A^vi^—Na1—O4	115.0 (4)	O4—As1—O3	103.3 (2)
O6B^vi^—Na1—O1	86.1 (4)	O6A—As2—O6A^xi^	78.0 (15)
O3^vii^—Na1—O1	165.2 (2)	O6A—As2—O5	125.7 (7)
O1^iii^—Na1—O1	81.7 (2)	O6A^xi^—As2—O5	112.6 (6)
O6A^vi^—Na1—O1	101.5 (5)	O6A—As2—O5^xi^	112.6 (6)
O4—Na1—O1	62.78 (17)	O6A^xi^—As2—O5^xi^	125.7 (7)
O6B^vi^—Na1—O4^vii^	130.4 (5)	O5—As2—O5^xi^	103.0 (3)
O3^vii^—Na1—O4^vii^	58.22 (16)	O6A—As2—O6B	28.8 (6)
O1^iii^—Na1—O4^vii^	126.1 (2)	O6A^xi^—As2—O6B	104.5 (12)
O6A^vi^—Na1—O4^vii^	110.9 (5)	O5—As2—O6B	104.3 (5)
O4—Na1—O4^vii^	61.4 (2)	O5^xi^—As2—O6B	104.8 (4)
O1—Na1—O4^vii^	123.09 (19)	O6A—As2—O6B^xi^	104.5 (12)
O6A—Na2—O6A^vi^	141.3 (12)	O6A^xi^—As2—O6B^xi^	28.8 (6)
O6A—Na2—O6B	21.3 (4)	O5—As2—O6B^xi^	104.8 (4)
O6A^vi^—Na2—O6B	160.8 (9)	O5^xi^—As2—O6B^xi^	104.3 (5)
O6A—Na2—O6B^vi^	160.8 (9)	O6B—As2—O6B^xi^	132.5 (10)
O6A^vi^—Na2—O6B^vi^	21.3 (4)	As1—O1—Na3	157.3 (3)
O6B—Na2—O6B^vi^	177.8 (8)	As1—O1—Na1^v^	118.2 (3)
O6A—Na2—O5^viii^	118.4 (6)	Na3—O1—Na1^v^	75.36 (17)
O6A^vi^—Na2—O5^viii^	94.8 (5)	As1—O1—Na1	91.9 (2)
O6B—Na2—O5^viii^	97.9 (4)	Na3—O1—Na1	71.66 (16)
O6B^vi^—Na2—O5^viii^	80.2 (3)	Na1^v^—O1—Na1	146.7 (3)
O6A—Na2—O5^ix^	94.8 (5)	As1—O1—Cs1	105.5 (2)
O6A^vi^—Na2—O5^ix^	118.4 (6)	Na3—O1—Cs1	90.6 (2)
O6B—Na2—O5^ix^	80.2 (3)	Na1^v^—O1—Cs1	94.33 (17)
O6B^vi^—Na2—O5^ix^	97.9 (4)	Na1—O1—Cs1	90.47 (16)
O5^viii^—Na2—O5^ix^	64.8 (3)	As1—O2—Hf2	147.4 (3)
O1^v^—Na3—O1^iii^	87.0 (3)	As1—O3—Hf1	130.0 (3)
O1^v^—Na3—O1	87.0 (3)	As1—O3—Na1^xvi^	108.8 (2)
O1^iii^—Na3—O1	87.0 (3)	Hf1—O3—Na1^xvi^	121.1 (2)
O1^v^—Na3—O6B^viii^	84.6 (2)	As1—O4—Hf2^xiv^	146.9 (3)
O1^iii^—Na3—O6B^viii^	170.0 (4)	As1—O4—Na1	95.3 (2)
O1—Na3—O6B^viii^	87.2 (2)	Hf2^xiv^—O4—Na1	110.6 (2)
O1^v^—Na3—O6B^x^	87.2 (2)	As1—O4—Na1^xvi^	86.8 (2)
O1^iii^—Na3—O6B^x^	84.5 (2)	Hf2^xiv^—O4—Na1^xvi^	95.70 (19)
O1—Na3—O6B^x^	170.0 (4)	Na1—O4—Na1^xvi^	122.6 (2)
O6B^viii^—Na3—O6B^x^	100.3 (3)	As2—O5—Hf1	137.1 (3)
O1^v^—Na3—O6B^vi^	170.0 (4)	As2—O5—Na2^xiii^	96.1 (2)
O1^iii^—Na3—O6B^vi^	87.2 (2)	Hf1—O5—Na2^xiii^	125.3 (2)
O1—Na3—O6B^vi^	84.6 (2)	As2—O6A—Na2	119.0 (8)
O6B^viii^—Na3—O6B^vi^	100.3 (3)	As2—O6A—Na1^vi^	116.5 (9)
O6B^x^—Na3—O6B^vi^	100.3 (3)	Na2—O6A—Na1^vi^	116.2 (7)
O6A^xi^—Na4—O6A^vi^	110.0 (4)	As2—O6A—Na4^xii^	146.0 (12)
O6A^xi^—Na4—O6A^xii^	110.0 (4)	Na2—O6A—Na4^xii^	74.0 (5)
O6A^vi^—Na4—O6A^xii^	110.0 (4)	Na1^vi^—O6A—Na4^xii^	77.5 (5)
O5^xiii^—Hf1—O5^ix^	92.17 (19)	As2—O6A—Na2^xvi^	83.9 (8)
O5^xiii^—Hf1—O5	92.17 (19)	Na2—O6A—Na2^xvi^	105.6 (8)
O5^ix^—Hf1—O5	92.17 (19)	Na1^vi^—O6A—Na2^xvi^	109.2 (6)
O5^xiii^—Hf1—O3^ix^	87.7 (2)	Na4^xii^—O6A—Na2^xvi^	62.2 (5)
O5^ix^—Hf1—O3^ix^	92.3 (2)	As2—O6B—Na1^vi^	120.8 (5)
O5—Hf1—O3^ix^	175.57 (19)	As2—O6B—Na2	103.8 (6)
O5^xiii^—Hf1—O3^xiii^	92.3 (2)	Na1^vi^—O6B—Na2	120.7 (4)
O5^ix^—Hf1—O3^xiii^	175.56 (19)	As2—O6B—Na3^xvii^	115.6 (6)
O5—Hf1—O3^xiii^	87.7 (2)	Na1^vi^—O6B—Na3^xvii^	77.3 (5)
O3^ix^—Hf1—O3^xiii^	87.89 (18)	Na2—O6B—Na3^xvii^	117.8 (5)
O5^xiii^—Hf1—O3	175.56 (19)		

Symmetry codes: (i) −*x*, −*y*, −*z*; (ii) *x*−*y*, *x*, −*z*; (iii) −*x*+*y*, −*x*, *z*; (iv) *y*, −*x*+*y*, −*z*; (v) −*y*, *x*−*y*, *z*; (vi) *x*−*y*+1/3, −*y*+2/3, −*z*+1/6; (vii) −*y*+1, *x*−*y*, *z*; (viii) *y*−2/3, *x*−1/3, −*z*+1/6; (ix) −*x*+*y*, −*x*+1, *z*; (x) −*x*+1/3, −*x*+*y*−1/3, −*z*+1/6; (xi) −*x*+4/3, −*x*+*y*+2/3, −*z*+1/6; (xii) *y*+1/3, *x*−1/3, −*z*+1/6; (xiii) −*y*+1, *x*−*y*+1, *z*; (xiv) −*x*+1, −*y*+1, −*z*; (xv) *y*, −*x*+*y*+1, −*z*; (xvi) −*x*+*y*+1, −*x*+1, *z*; (xvii) *y*+1/3, *x*+2/3, −*z*+1/6.
